# Epidemiological characteristics of acute viral and mycoplasma respiratory infections in Yongzhou, China: a retrospective descriptive study

**DOI:** 10.3389/fpubh.2025.1614985

**Published:** 2025-07-17

**Authors:** Ying Jiang, Junyan Lu, Zhengrong Tan, Hanmei Wan, Min Tang, Jiani Li, Tao Zhou, Wenlong Tang

**Affiliations:** ^1^Department of Center for Precision Medicine, The Central Hospital of Yongzhou, Yongzhou, China; ^2^Department of Center for Precision Medicine, Yongzhou Hospital Affiliated to University of South China, Yongzhou, China

**Keywords:** acute respiratory infections, respiratory pathogens, epidemic characteristics, COVID-19, co-infections

## Abstract

**Background:**

Acute respiratory infections (ARIs) are major global public health concerns. Understanding the epidemiological characteristics and evolution patterns of respiratory pathogens before and after the coronavirus disease 2019 (COVID-19) pandemic is crucial for disease control and prevention. This study identified the epidemiological characteristics and pathogen distribution in hospitalized patients with ARIs in Yongzhou, China.

**Methods:**

A retrospective analysis was conducted on 10,728 hospitalized patients with ARIs at a tertiary hospital in Yongzhou, China, from January 2019 to June 2024. Respiratory specimens were tested using standardized protocols including multiplex real-time PCR for detecting common respiratory pathogens (FluA and FluB, RSV, ADV, HRV, and MP) and next-generation sequencing for additional pathogen identification when clinically indicated. Statistical analyses included descriptive statistics for demographic and clinical characteristics, and chi-square tests for comparing categorical variables across different age groups, seasons, and time periods.

**Results:**

Overall, 43.12% (4,626/10,728) of samples were positive for at least one pathogen. The most frequently detected pathogens were FluA (11.95%), RSV (9.61%), and MP (8.73%). RSV primarily affected children under 5 years (38.63% of RSV cases), while SARS-CoV-2 showed higher detection rates in older adult populations (64.81% of COVID-19 cases). Co-infections were found in 23.76% (1,099/4,626) of positive samples, with preschool children (2–5 years) showing the highest rate at 32.58%. MP (*n* = 398, 36.21% of all co-infections) with HRV (*n* = 338, 30.76% of all co-infections) were the most frequently detected pathogens in co-infections. Significant seasonal variations were observed, with winter showing the highest pathogen detection rates (49.04%, *p* < 0.001), and seasonal patterns changed notably during the COVID-19 pandemic. The frequency and patterns of co-infections showed marked differences across pre-pandemic, pandemic, and post-pandemic (January 2023–June 2024) periods. Co-infections occurred in 1,209 cases, with significant differences across study periods: 20.6% in pre-pandemic, 0.6% during pandemic, and 78.8% in post-pandemic periods (*p* < 0.001).

**Conclusion:**

This study revealed distinct age-dependent and seasonal distribution patterns of respiratory pathogens in Yongzhou, China. The significant changes in pathogen circulation dynamics before, during, and after the COVID-19 pandemic highlight the importance of continuous surveillance of respiratory viruses. These findings provide valuable insights for optimizing local ARI prevention and treatment services.

## 1 Introduction

Acute Respiratory Infection (ARI) represents a critical global public health challenge, accounting for millions of emergency visits and hospitalizations annually (1, 2). According to the World Health Organization’s 2019 data, ARI, particularly Lower Respiratory Tract Infection (LRTI), has emerged as a leading cause of mortality and morbidity worldwide, posing substantial challenges to public health systems (3, 4). The high infectivity and rapid transmission characteristics of respiratory pathogens frequently result in regional or global disease outbreaks, underscoring the urgency of addressing this health concern (5).

More than 200 distinct viruses are known to cause ARI with varying degrees of severity (6). These respiratory viruses, originating from diverse viral families, exhibit remarkable variations in their genomic features, host preferences, pathogenic mechanisms, seasonal patterns, and transmission dynamics (2). Notable examples include influenza viruses (Flu), characterized by significant antigenic variations leading to severe infections; coronaviruses (CoV) such as Severe Acute Respiratory Syndrome Coronavirus 1 (SARS-CoV-1) and Middle East Respiratory Syndrome Coronavirus (MERS-CoV), which triggered global epidemics with mortality rates reaching 34.4%; Adenoviruses (ADV) and respiratory syncytial virus (RSV) are major pathogens affecting pediatric respiratory health, manifesting as various conditions ranging from pharyngitis and bronchitis to pneumonia and other respiratory tract infections; and human metapneumovirus (hMPV) emerging as a primary cause of anaerobic pneumonia in infants (7–13). A comprehensive understanding of these pathogens’ epidemiological characteristics is fundamental to mitigating their public health impact.

The Coronavirus Disease 2019 (COVID-19) outbreak in late 2019 generated millions of respiratory emergency cases and prompted the Chinese government to implement comprehensive control measures, including universal mask-wearing, stringent hand hygiene protocols, isolation of infected individuals, and mass vaccination programs. These interventions may have fundamentally altered the transmission patterns of common respiratory pathogens. Given the limited development of vaccines against respiratory viral infections, an in-depth study of ARI’s viral etiology remains crucial for effective epidemic prevention and control. This study presents a systematic analysis of clinical manifestations and demographic characteristics among hospitalized patients (both children and adults) with acute respiratory infections in Yongzhou from 2019 to 2024, elucidating the epidemiological patterns of respiratory pathogens in the region. The findings provide essential baseline data for ARI prevention and control in Yongzhou, thoroughly examine the impact of the COVID-19 epidemic and associated control measures on common respiratory pathogen transmission, offering valuable insights for future clinical management strategies.

## 2 Materials and methods

### 2.1 Study subjects and data collection

This study employed a retrospective research design, collecting and analyzing data from ARTI patients hospitalized at Yongzhou Central Hospital from January 2019 to June 2024.

Yongzhou Central Hospital is a comprehensive tertiary Grade A hospital. The hospital has approximately 2,100 beds, serves as the regional medical and healthcare center for Yongzhou City, with responsibility for emergency response to public health emergencies and critical care services for the city’s 6.45 million population. As the primary referral center for acute respiratory infections in the region, the hospital’s patient population is representative of the broader epidemiological patterns in this area of Hunan Province, China.

Patient selection criteria were as follows. Inclusion criteria included:

(1) Clinical manifestations: fever, accompanied by at least one of the following symptoms: cough, nasal congestion, rhinorrhea, sore throat, sputum production, or headache.

(2) Diagnostic criteria: Based on discharge diagnoses recorded in the hospital’s electronic medical record system, including but not limited to bronchopneumonia, influenza, viral pneumonia, severe pneumonia, obstructive pneumonia, acute upper respiratory tract infection, viral cold, influenza with pneumonia, type H1N1 influenza (pneumonia), rhinovirus infection (acute bronchitis), parainfluenza virus infection, respiratory syncytial virus infection (pneumonia, acute bronchitis), mycoplasma pneumonia, and Severe Acute Respiratory Syndrome Coronavirus 2 (SARS-CoV-2) infection.

Exclusion criteria: (1) Patients with unclear diagnosis: Patients discharged without a definitive respiratory infection diagnosis or with conflicting diagnostic information.

(2) Cases with exclusively typical bacterial infections: Patients with respiratory infections caused solely by typical bacterial pathogens (e.g., *Streptococcus pneumoniae*, *Haemophilus influenzae*) confirmed by bacterial culture or antigen detection, without concurrent detection of viral pathogens or MP.

### 2.2 Pathogenic detection methods

Throughout the study period (2019–2024), we employed standardized diagnostic testing protocols for respiratory pathogens, though detection technologies evolved over time. To maintain consistency in data comparison across years, we established a comprehensive testing framework that included the following methods:

(1) Influenza A/B antigen detection: we used the influenza A/B virus antigen detection reagent (colloidal gold immunochromatographic assay; Wondfo Biotech Co., Ltd., Guangzhou, China) for qualitative detection based on the double-antibody sandwich method. Standardized pharyngeal swab collection was completed within 24 h of admission for all patients, with sampling sites at the tonsils and posterior pharyngeal wall. This method was consistently applied throughout the study period.

(2) Multiple respiratory pathogen nucleic acid detection: we used Sansure Biotech’s nucleic acid extraction kit and respiratory pathogen nucleic acid detection kit (Sansure Biotech Inc., Changsha, China) for simultaneous detection of FluA, FluB, HRV, RSV, ADV, and MP using multiplex real-time fluorescent quantitative PCR technology. This RT-PCR based assay is a clinically validated molecular diagnostic method that demonstrates high sensitivity and specificity for respiratory pathogen detection, with the capability of simultaneous identification of six respiratory pathogens in a single test. The multiplex PCR design enables efficient differential diagnosis and supports clinical decision-making with rapid turnaround time. This constituted our core testing panel that was applied to all patients meeting inclusion criteria. Additional pathogens including parainfluenza virus (PIV) and hMPV were identified through the next-generation sequencing methods described below.

(3) Next-generation sequencing technology: targeted Next-Generation Sequencing (tNGS) and Metagenomic Next-Generation Sequencing (mNGS) were performed by Guangzhou KingMed Medical Testing Center Co., Ltd. and Shenzhen BGI Genomics Co., Ltd., respectively. These methods were employed for patients with severe clinical manifestations or those with negative results from conventional testing but strong clinical suspicion of viral infection. The standard protocols established by these reference laboratories remained consistent throughout the study period. SARS-CoV-2 testing was conducted using specific RT-PCR assays according to national diagnostic protocols, which were incorporated into our testing panels from January 2020 onward.

Next,-generation sequencing technology: for cases requiring advanced molecular diagnostics, we employed either Targeted Next-Generation Sequencing (tNGS) performed by Guangzhou KingMed Medical Testing Center Co., Ltd., or Metagenomic Next-Generation Sequencing (mNGS) conducted by Shenzhen BGI Genomics Co., Ltd. The choice between tNGS and mNGS was made on a case-by-case basis, with tNGS typically used as the first-line approach for suspected viral respiratory infections, while mNGS was reserved for cases where broader pathogen screening was clinically indicated. These advanced sequencing methods were selectively employed for patients with severe clinical manifestations or those with negative results from conventional RT-PCR testing but with strong clinical suspicion of viral infection.

All laboratory procedures followed standardized protocols with regular quality control and validation procedures to ensure methodological consistency throughout the study period. SARS-CoV-2 testing was incorporated into our diagnostic panels from January 2020 onward using specific RT-PCR assays according to national diagnostic protocols.

### 2.3 Statistical analysis

Data processing, statistical analysis, and graphic generation were written in R version 4.4.0. Categorical variables were expressed as number (percentage) [n (%)].

Age groups were defined as follows: 0–1 years (0 to <1 year), 1–2 years (1 to <2 years), 2–5 years (2 to <5 years), 5–18 years (5 to <18 years), 18–60 years (18 to <60 years), and ≥60 years (60 years and above). All age ranges are inclusive of the lower bound and exclusive of the upper bound, except for the eldest group which includes 60 years and above.

Descriptive statistical methods were used to analyze seasonal distribution, age distribution, and infection rate characteristics of respiratory viruses. Chi-square test (*χ*^2^ test) was used to compare differences in virus positivity between different age groups, genders, and clinical characteristics. All statistical tests were two-sided, with *p* < 0.05 considered statistically significant.

## 3 Results

### 3.1 Demographic characteristics and clinical manifestations

The demographic data are shown in Table 1. A total of 10,728 samples were collected, with males and females representing 62.23 and 37.77% of the population, respectively. More than 78.8% of patients were aged under 18 years, with the majority (63.84%) being patients under 5 years. The main clinical manifestations were cough and fever, followed by tachypnea, wheezing, and sputum production.

**Table 1:** Clinical characteristics of hospitalized patients with ARIs.

Items	Classifications	Number of cases	Proportion (%)
Gender	Male	6,676	62.23
	Female	4,502	37.77
Age	0 ~ 1	3,148	29.34
	1 ~ 2	1,376	12.83
	2 ~ 5	2,325	21.67
	5 ~ 18	1,605	14.96
	18 ~ 60	841	7.84
	≥60	1,433	13.36
Clinical diagnosis	Fever	6,449	60.11
	Cough	8,368	78.00
	Sneezing	100	0.93
	Nasal discharge	446	4.16
	Sore throat	89	0.83
	Tachypnea	903	8.42
	Nasal congestion	208	1.94
	Wheezing	824	7.68
	Coughing up phlegm	773	7.21
	Stridor	338	3.15

### 3.2 Overall positive detection

**Figure 1:**
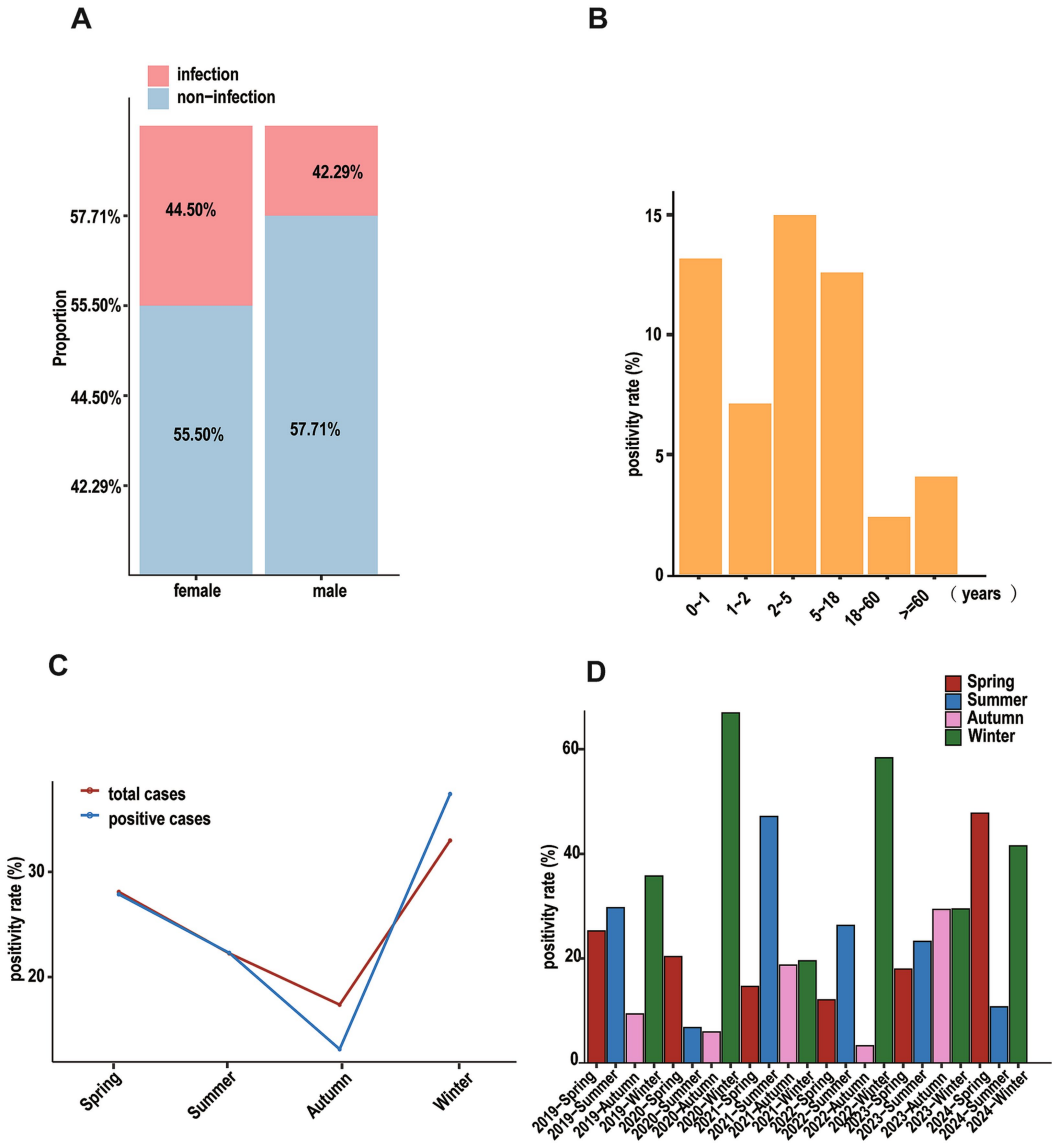
Positive cases of patients based on different dimensions. **(A)** Stacked diagram of patients for different genders. **(B)** That of patients based on ages. **(C)** That of patients based on seasons, the red line represents all hospitalized patients in that season, and the blue line represents the positive patients in that season. **(D)** Bar graph showing the positivity rate of patients in different seasons annually.

Among hospitalized patients, 4,626 cases (43.12%) tested positive for at least one respiratory pathogen. Males (42.29%) and females (44.50%) showed statistically significant different detection rates (*χ*^2^ = 4.93, *p* = 0.026) (Figure 1A). Age-stratified analysis demonstrated that preschool children (≤5 years) had the highest positivity rate (26.68%), whereas the lowest rates were observed in adults aged 18–60 years (Figure 1B), significant differences were observed among age groups (*χ*^2^ = 609.75, *p* < 0.001).

There were distinct seasonal variations in pathogen detection rates: winter showed the highest positivity (37.53%), followed by spring (27.76%), summer (22.05%), and autumn (12.67%) (Figure 1C). Annual comparisons revealed distinct patterns in seasonal distribution (Figure 1D). A typical winter-dominant pattern was observed in 2019, 2020, and 2022, with rates decreasing through summer, spring, and autumn. In contrast, 2021 exhibited an unusual pattern with peak positivity in summer, followed by winter and autumn, with spring showing the lowest rates.

### 3.3 Pathogen spectrum and age patterns

**Figure 2:**
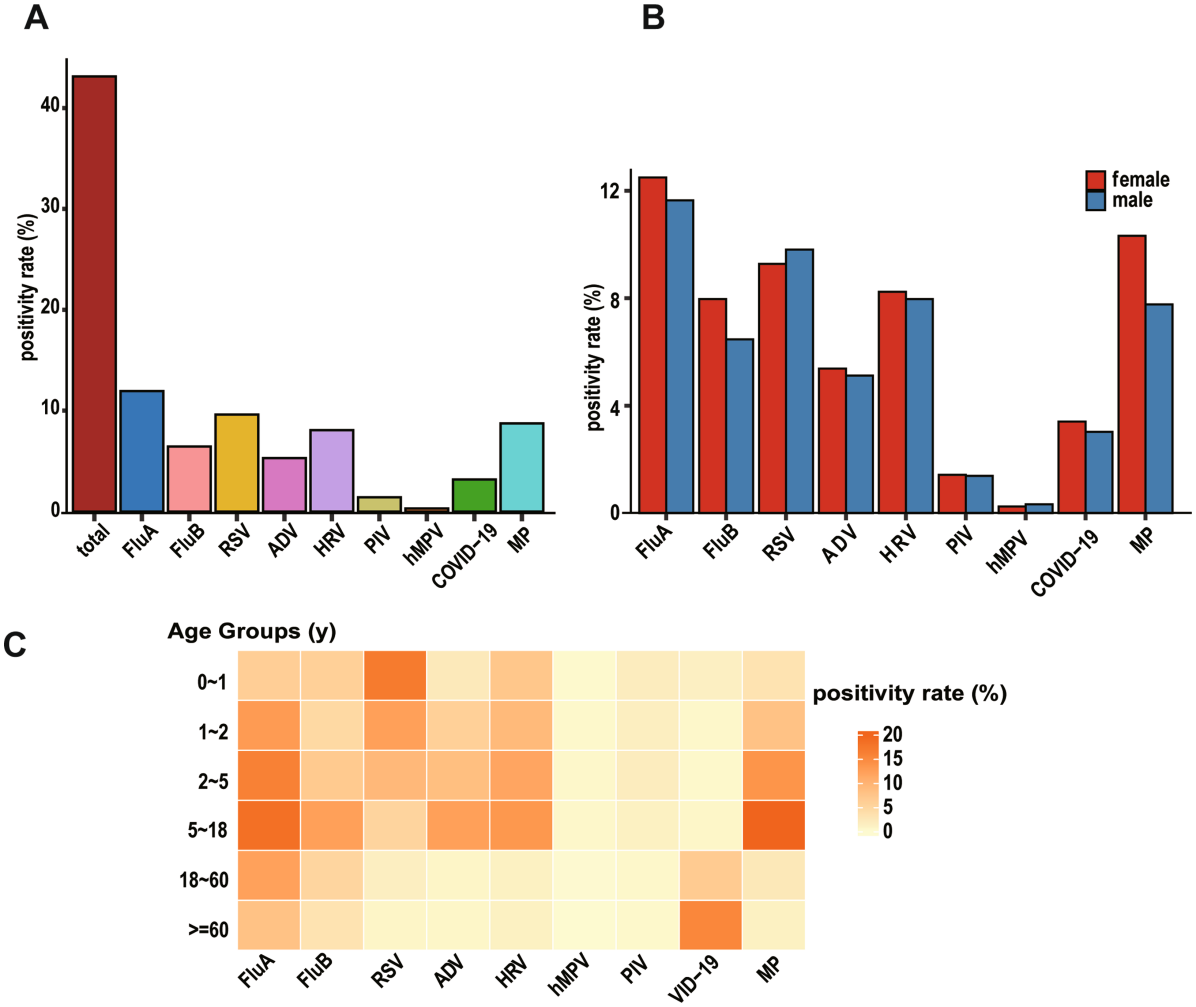
Identification of viral etiologies based on virus-positive patients with ARIs. **(A)** Overall positivity rate of all viruses (“total” means overall positivity rate of all patients). **(B)** Bar graph showing the positivity rate by gender for each virus. **(C)** Pheatmap displaying positivity rate of viral infections across different age groups, with each row representing an age group and each column representing a virus.

FluA was most prevalent (11.95%) among detected pathogens, followed by RSV (9.61%), MP (8.73%), HRV (8.07%), FluB (6.45%), ADV (5.30%), SARS-CoV-2 (3.18%), PIV (1.41%), and hMPV (0.30%) (Figure 2A). The detection rates of respiratory pathogens showed comparable distributions between genders (Figure 2B).

Pathogen distribution patterns varied significantly among age groups. RSV showed a distinctive age-dependent pattern, with detection rates highest in infants aged 0–1 year (16.96%) and young toddlers aged 1–2 years (12.43%), followed by a progressive decline to 9.25% in preschoolers (2–5 years), 5.23% in school-age children and adolescents (5–18 years), 1.43% in adults (18–60 years), and 0.70% in the older adult (≥60 years). Notably, infants under 1 year of age constituted 52.05% of all RSV-positive cases, highlighting RSV’s strong age tropism with infection risk significantly decreasing with advancing age.

The PIV infections demonstrated similar age-specific distribution, with the highest burden in young children: infants (0–1 year) and preschoolers (2–5 years) together accounted for 66.89% of all PIV-positive cases. Peak detection rates for both HRV and hMPV were observed in preschool children (2–5 years), representing 31.52 and 32.35% of their respective positive cases. In contrast, SARS-CoV-2 infections displayed a distinct older adult predominance, with patients aged ≥ 60 years constituting 64.81% of all COVID-19 positive cases. ADV and FluA were predominantly detected in children and adolescents (2–18 years), while FluB exhibited high detection rates in both infants (0–1 year) and the 2–18 years age group (Figure 2C). The detailed positive data are shown in Supplementary Table S1.

### 3.4 Seasonality distribution of pathogens

**Figure 3:**
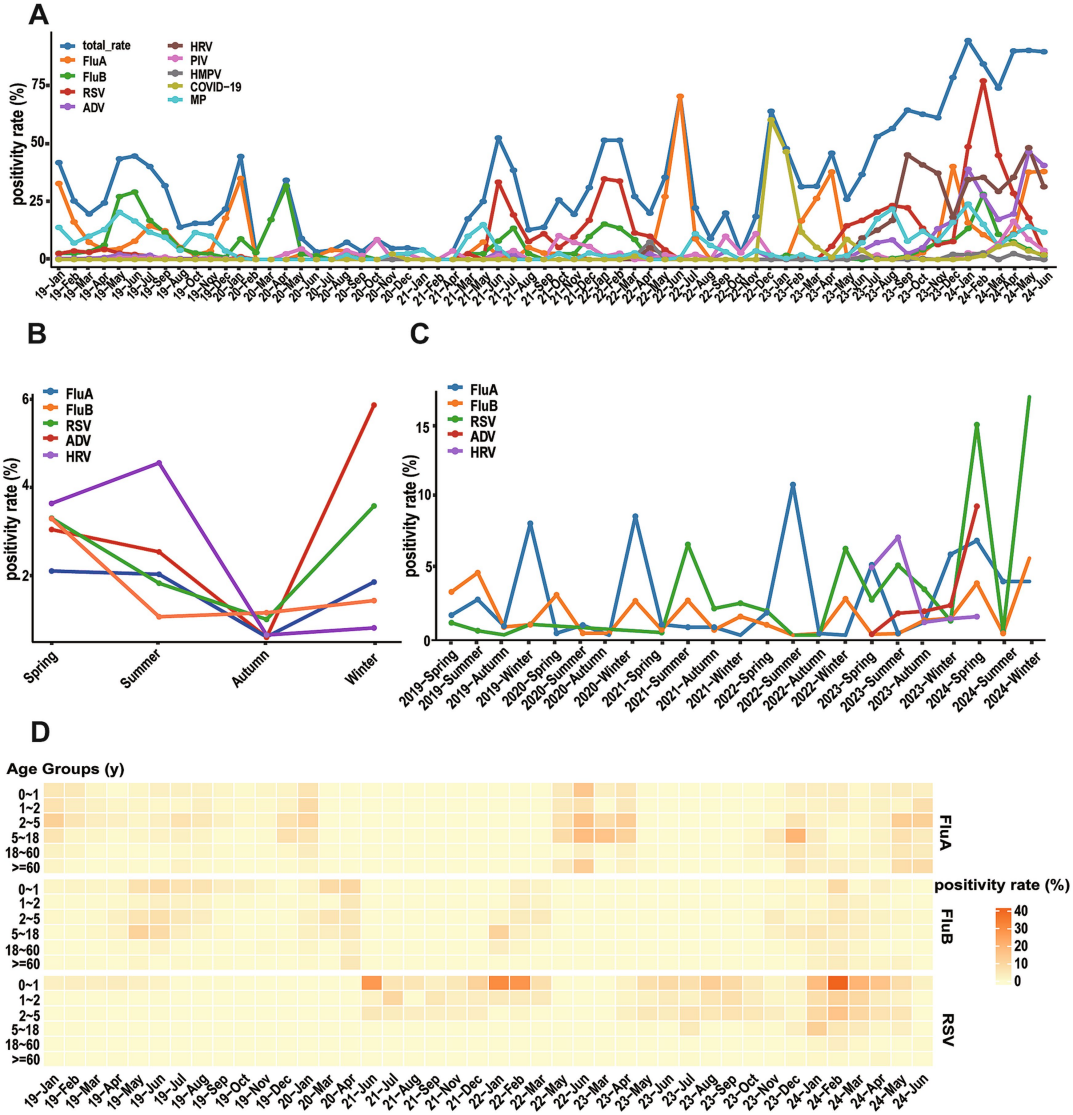
Seasonal distribution (months) of different respiratory viruses. **(A)** Monthly positivity rate of different respiratory viruses and total from January 2019 to June 2024. **(B)** Total seasonal positive rates for five major viruses. **(C)** Annual seasonal positivity rate for five major viruses. **(D)** Peatmap illustrates positivity rate of FluA, FluB, and RSV across different age groups and months.

During the study period, respiratory pathogens were detected in all months except February 2021, with monthly overall positivity rates fluctuating between 2.99 and 92.51%. PIV, hMPV, MP, and SARS-CoV-2 showed relatively stable epidemic trends, though most pathogens showed increased detection rates in June and July (Figure 3A).

Although the overall detection rate of acute respiratory infections peaked in summer, each virus exhibited distinct seasonal distribution patterns (Figure 3B). FluA exhibited maximum prevalence in winter with a detection rate of 42.80%, while reaching its nadir in autumn. RSV displayed a characteristic bimodal distribution with prominent peaks in winter and spring, reaching detection rates of 48.75 and 25.28%, respectively. The detection rates of ADV and FluB increased during winter and spring, remaining relatively low in summer and autumn. In contrast, HRV demonstrated peak prevalence during summer, with diminished activity during autumn and winter. These divergent seasonal patterns reflect the unique transmission dynamics of each pathogen. Statistical analysis confirmed winter as the highest-risk season (49.04%) with significant differences across all seasons (*χ*^2^ = 134.67, *p* < 0.001).

Further analysis revealed that significant changes in seasonal distribution patterns of some pathogens before and after the COVID-19 pandemic (Figure 3C). FluA demonstrated distinct temporal changes, with epidemic peaks primarily occurring in winter during 2019–2020, shifting to summer in 2022, and evolving into spring–winter double peaks in 2023. The seasonal pattern of FluB showed more slightly variations: a summer peak in 2019, spring–winter peaks in 2020, a return to summer predominance in 2021, and winter concentration during 2022–2023. RSV maintained relatively low levels in 2019–2020, followed by prominent epidemic peaks in summer 2021, winter 2022, and summer 2023. Notably, detection rates of these three viruses all showed an upward trend in 2024 (Figure 3D).

### 3.5 Co-infection

For this study, we defined co-infection as the simultaneous detection of two or more respiratory pathogens in a single patient sample through our standardized testing protocols, which included multiplex PCR or next-generation sequencing results. Among 4,626 positive cases, 1,099 cases (23.76%) of multiple pathogen infections were detected. Male detection rate (59.78%) was higher than female (40.22%), but the difference was not statistically significant (*χ*^2^ = 3.84, *p* = 0.050). However, significant differences were observed in co-infection rates across age groups (*χ*^2^ = 132.1, *p* < 0.001), with preschool children (2–5 years) showing the highest rate at 32.58%. Specifically, 929 cases (84.53%) demonstrated dual infections, 147 cases (13.38%) had triple infections, 19 cases (1.73%) showed quadruple infections, and 4 cases (0.36%) presented with quintuple infections.

**Figure 4:**
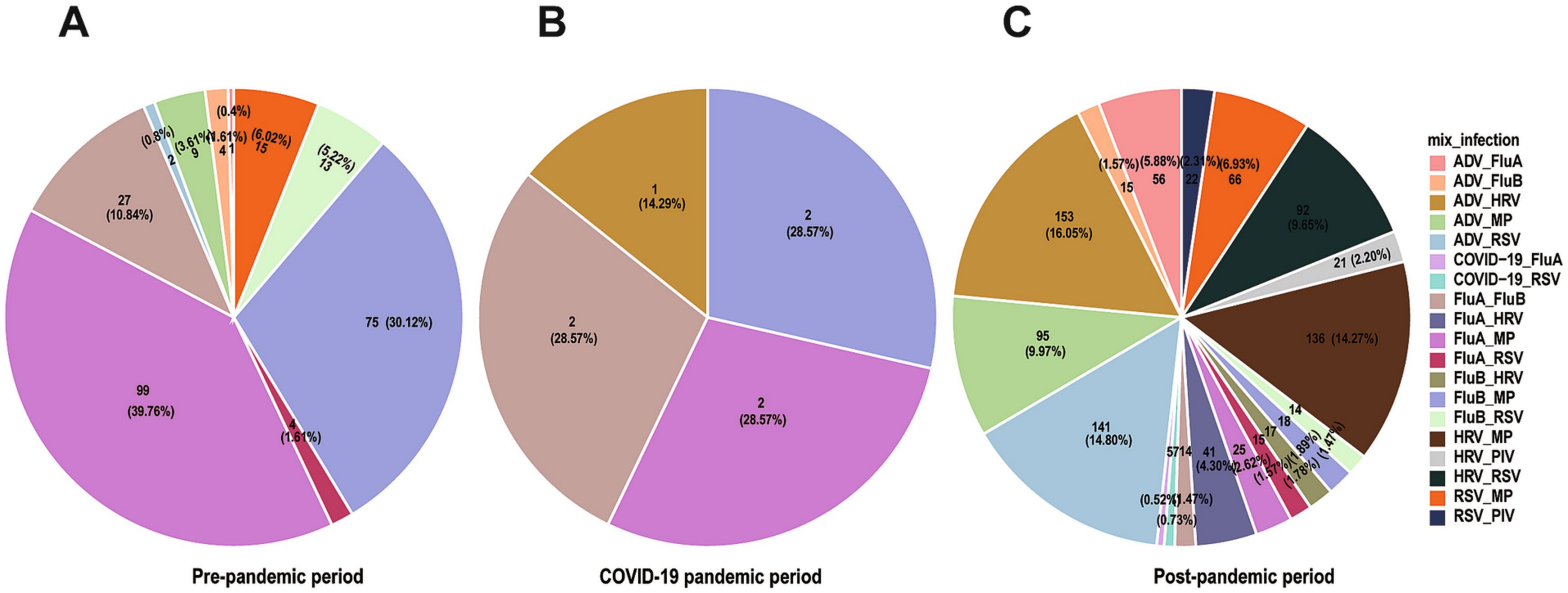
Co-infection patterns across different time periods. Pie charts showing proportions of different co-infection patterns, with **(A–C)** representing pre-epidemic period, during the epidemic, and post-epidemic.

Analysis of co-infection combinations showed MP (*n* = 398, 36.21% of all co-infections) with HRV (*n* = 338, 30.76% of all co-infections) were the most frequently detected pathogens in co-infections, followed by ADV (*n* = 321, 29.21%), RSV (*n* = 279, 25.39%) and FluA (*n* = 255, 23.21%). The frequency of co-infections showed significant differences across the three study periods: 249 cases (20.6%) in the pre-pandemic period, 7 cases (0.6%) during the pandemic period, and 953 cases (78.8%) in the post-pandemic period (*χ*^2^ = 1198.37, *p* < 0.001) (Figures 4A–C). During the pandemic control period, the incidence of co-infections decreased markedly (*χ*^2^ = 45.30, *p* < 0.001), suggesting the effectiveness of these interventions in suppressing respiratory virus transmission. The predominant co-infection patterns before the pandemic primarily involved combinations of influenza viruses with MP, as well as co-infections of FluA and FluB. However, following the relaxation of control measures, ADV-HRV (*n* = 154, 12.74%), ADV-RSV (*n* = 143, 11.83%), and HRV-MP (*n* = 136, 11.25%) combinations emerged as the dominant patterns. SARS-CoV-2 co-infections were rare (*n* = 12), including COVID-19 with FluA (*n* = 5) and COVID-19 with RSV (*n* = 7), likely due to effective pandemic control measures during the study period. This shift suggests that co-infection patterns may be significantly influenced by the prevailing epidemiological context, which has important implications for clinical diagnosis and treatment.

## 4 Discussion

Acute respiratory infections represent a significant public health and economic burden; understanding the etiology and epidemiology of respiratory viruses is imperative for ARI prevention and control (14, 15). This study conducted a systematic analysis of age distribution patterns and seasonal variations of respiratory pathogens in the Yongzhou region from January 2019 to June 2024. The results showed that 43.12% of specimens tested positive for at least one respiratory pathogen; however, this detection rate may have been influenced by factors such as the spectrum of pathogens tested, detection methodologies, and study population characteristics.

Our study identified the predominant respiratory pathogens as FluA, FluB, RSV, ADV, and HRV, consistent with previous research findings (15, 16). While influenza viruses demonstrated the highest detection rates among ARI patients, their positivity rates declined markedly during the COVID-19 pandemic, with FluA dropping from 12.64 to 7.92% and FluB from 8.9 to 4.56%. This trend aligned with reports from southern China (17). Concurrently, RSV infection rates decreased markedly during the early pandemic and strict control period but showed significant rebounds after control measures were gradually relaxed, particularly in the second and third years of the COVID-19 pandemic, even exceeding pre-pandemic levels. This resurgence likely resulted from weakened population immunity barriers and increased viral transmission following the relaxation of social restrictions, which demonstrates the effectiveness of pandemic control measures in reducing respiratory virus transmission.

Unlike previous studies primarily focusing on pediatric populations, this study included hospitalized patients across all age groups (18). Results showed that children under 5 years had the highest risk of RSV infection, further confirming the age-specific nature of RSV infections, likely associated with children’s relatively weak innate immune responses (18–22). Additionally, SARS-CoV-2 showed higher detection rates among adults and older adult populations, consistent with previous research findings, suggesting age may be one of the important factors affecting respiratory virus infection spectrum (23).

Our study identified statistically significant gender differences in overall pathogen detection rates, which differs from several published reports that found no gender disparities (6, 21, 24, 25). This discrepancy may stem from variations in sample size, demographic characteristics, or pathogen prevalence across study populations. Regarding seasonal distribution, overall detection rates were lowest in autumn, possibly related to the region’s specific climatic characteristics and population behavior patterns. Influenza viruses, particularly FluA, as important respiratory pathogens, reached epidemic peaks during spring and winter in our study. This pattern differs from some previous studies, suggesting that geographical location and climatic factors may influence pathogen circulation patterns.

Evidence suggests that respiratory infections may be caused by multiple pathogens (21, 26–28). In our study, 10.24% of hospitalized patients exhibited co-infections, with 23.76% of positive cases showing multiple pathogens. Some studies suggest potential interference between different pathogens, though the precise mechanisms of these interactions remain unclear (29, 30). Virus-induced interferons and other cytokines may produce cross-protective effects, a hypothesis warranting further investigation (29, 31). We found that ADV, HRV, and RSV were the most common pathogens in co-infections, with viral co-infections predominantly occurring in children under 5 years. The findings indicate that pathogen detection rates in respiratory infections are closely associated with patient age, as age influences immune status, viral exposure opportunities, and other lifestyle factors. Generally, young children and older adult individuals are more susceptible to viral infections due to their compromised immune systems (20, 32–34). Deep understanding of interactions between pathogens has important guiding significance for grasping respiratory infection epidemic patterns and optimizing clinical treatment strategies.

Several limitations should be acknowledged. First, the analysis focused only on major respiratory viruses and MP, excluding bacterial pathogens and chlamydia, thus providing an incomplete picture of respiratory infection epidemiology in the region. Second, the study’s generalizability is limited by its retrospective nature and reliance on hospitalized patients from a single medical center, potentially introducing selection bias as outpatient cases were not included. Third, while we maintained consistent testing protocols throughout the study period, the evolution of detection technologies and the introduction of SARS-CoV-2 testing from 2020 onward may have influenced detection rates. In addition, we did not perform influenza subtyping or lineage determination, limiting assessment of anthropozoonotic potential and circulation patterns. Future studies should incorporate comprehensive influenza subtyping for detailed epidemiological characterization. Last, our retrospective study did not collect demographic risk factors, occupational data, or socioeconomic information that might explain these gender differences. Nevertheless, these findings provide valuable epidemiological insights into ARI populations in the Yongzhou region. Large-scale, multi-center prospective studies are warranted to advance our understanding of respiratory pathogen epidemiology and guide evidence-based control measures.

## 5 Conclusion

In conclusion, our study investigated the epidemiological characteristics and temporal distribution of respiratory pathogens among hospitalized patients in Yongzhou, China, from 2019 to 2024, with particular attention to age-specific patterns and seasonal variations. The significant changes in pathogen circulation dynamics before, during, and after the COVID-19 pandemic highlight the importance of continuous surveillance of respiratory viruses. Such longitudinal monitoring is essential for understanding the evolving landscape of respiratory infections, especially in the context of major public health events. These findings provide valuable insights for optimizing diagnostic approaches and developing targeted prevention strategies, ultimately contributing to more effective control of respiratory viral infections in the region.

## Data Availability

The datasets used and/or analyzed during the current study are not publicly available due to institutional policies restricting the sharing of sensitive health data. However, de-identified data may be made available upon reasonable request and with permission from the ethics committee and the institution.
