# Supplementary material for: Epidemiological characteristics of acute viral and mycoplasma respiratory infections in Yongzhou, China: a retrospective descriptive study
Source: Front Public Health. 2025 Jul 17;13:1614985. doi: 10.3389/fpubh.2025.1614985 (PMC12310726; doi:10.3389/fpubh.2025.1614985)
Supplement: Supplementary file 1 [file Table_1.DOCX]

Supplementary Material

# Supplementary Figures and Tables

## Supplementary Tables

**Table S1 Age distribution of viruses from inpatients with ARIs**

| Age | Positive numbers (n) | | | | | | | | |
| --- | --- | --- | --- | --- | --- | --- | --- | --- | --- |
|  | FluA | FluB | RSV | ADV | HRV | PIV | HMPV | COVID-19 | MP |
| 0~1 | 193 | 184 | 534 | 72 | 227 | 57 | 8 | 39 | 99 |
| 1~2 | 180 | 62 | 171 | 81 | 127 | 21 | 5 | 6 | 111 |
| 2~5 | 376 | 160 | 215 | 200 | 273 | 42 | 12 | 9 | 321 |
| 5~18 | 297 | 199 | 84 | 199 | 215 | 17 | 8 | 9 | 323 |
| 18~60 | 104 | 43 | 12 | 6 | 9 | 5 | 1 | 57 | 20 |
| ≥60 | 116 | 44 | 10 | 8 | 15 | 6 | 1 | 221 | 16 |
